# Social messaging for global health: lessons from Haiti

**DOI:** 10.7189/jogh.09.010308

**Published:** 2019-06

**Authors:** Shada A Rouhani, Regan H Marsh, Linda Rimpel, Marie Cassandre Edmond, Marc Julmisse, Keegan A Checkett

**Affiliations:** 1 Department of Emergency Medicine, Brigham and Women’s Hospital, Boston, Massachusetts, USA; 2 Department of Emergency Medicine, Harvard Medical School, Boston, Massachusetts, USA; 3 Hôpital Universitaire de Mirebalais, Mirebalais, Haiti; 4 Partners In Health, Boston, Massachusetts, USA; 5 Department of Emergency Medicine, University of Chicago, Chicago, Ilinois, USA

In September 2016, Hurricane Matthew, a Category 4 storm, struck southern Haiti, an impoverished region home to 1.5 million people. Disturbingly, 24 hours later, the western half of the peninsula remained eerily silent. Communication lines were cut: bridges had washed away, roads were flooded or destroyed, and cellular service was virtually non-existent. As relief organizations attempted to assess damage, phone call after phone call went unanswered. St. Boniface Haiti Foundation (SBHF) ultimately made contact using a different tool: WhatsApp. While weak and inconsistent signals prevented voice calls, WhatsApp messages and replies were automatically delivered whenever signal allowed, allowing effective needs assessments. Information received helped SBHF assess the damage, share information with other organizations, and engender a timely, cooperative, organized, and appropriate relief effort.

Disaster response is only one way social messaging technology is impacting health care delivery in resource-limited settings. Over the past three years, our teams at Hôpital Universitaire de Mirebalais (HUM), a Partners In Health-supported academic hospital in central Haiti that collaborates with SBHF – have increasingly integrated social messaging into our operations. We present here our use of WhatsApp to improve health care delivery and education in Haiti and discuss considerations for successful use of social messaging in global health.

## SOCIAL MESSAGING

Social messaging applications (**Table 1**) allow users to communicate directly with one or more individuals [2]. Messages are targeted to specific groups of people, unlike social media which broadly transmits communications. Most applications use minimal data to transmit messages, allowing affordable use on cellular networks. Most also function over WiFi where available. Globally, social messaging’s popularity has increased and, by some measures, surpassed traditional social networking sites such as Facebook [3]. In resource-limited settings, social messaging has grown as smart phone technology has become more prevalent and affordable [4,5].

**Table 1:** Comparison of select features among select social messaging platforms. Platforms may have other features such as gaming, payments, or location services not detailed here

	Information		Features & Details					
	Monthly active users* (millions)	**Platforms**	**End-to-end encryption**	**Group messaging**	**Calling**	**Voice messages**	**Photo / video sharing**	**Document / file sharing**
**Whatsapp**	1,500	Android, iOS, Windows Phone, Web	Yes, by default	Yes	Voice and Video	Yes	Yes	Yes
**Facebook Messenger**	1,300	Android, iOS, Windows Phone, Web	Yes, must specifically enable	Yes	Voice and Video	Yes	Yes	Yes
**WeChat**	1,000	Android, iOS, BlackBerry, Windows Phone, Symbian, Web	No	Yes	Voice and Video	Yes	Yes	Yes
**QQ Mobile**	783	Android, iOS, Windows, OSX, Web	No	Yes	Voice and Video	Yes	Yes	Yes
**Skype**	300	Windows, Xbox, OSX, Linux, Android, iOS, Amazon Kindle/Fire	Yes, using “Private Conversations”	Yes	Voice and Video	Yes	Yes	Yes
**Viber**	260	Android, iOS, Windows, Linux, OSX	Yes	Yes	Voice and Video	Yes	Yes	Yes
**Snapchat**	255	Android, iOS	No	Yes, deleted by default after set timeframe	Voice and Video	Yes	Yes, deleted by default after set timeframe	No
**LINE**	203	Android, BlackBerry, iOS, Web, Windows, OSX	Yes, default for messaging, location share, voice & video calls	Yes	Voice and Video	Yes	Yes	No

*Average monthly users as of April 2018, as reported by Statistica [1].

Mobile technology and text messaging are recognized as promising ways to improve patient education, follow-up, and outcomes [6,7]. However, less attention has been paid to how social messaging applications can improve health care delivery, medical education, and emergency response. Social messaging allows real-time group responses and transmission of images and videos without the per message charges associated with text or multimedia messaging. For hospitals in resource-limited settings, pager technology is often prohibitively expensive, leaving a communication gap social messaging can fill. Our experience primarily has been with WhatsApp, because it is free, uses default end-to-end encryption, supports photo and video, and is already popular in Haiti, but the benefits likely apply across platforms with similar features.

Recently, several small studies have suggested that WhatsApp can facilitate local and remote patient consultation [8-10] and improve provider education [11]. Preliminary data indicate that mobile technologies including WhatsApp facilitate health, security and information access in refugee settings [12]. Additionally, our team has utilized social messaging to improve hospital operations, develop leadership skills, and create new educational opportunities.

**Figure:**
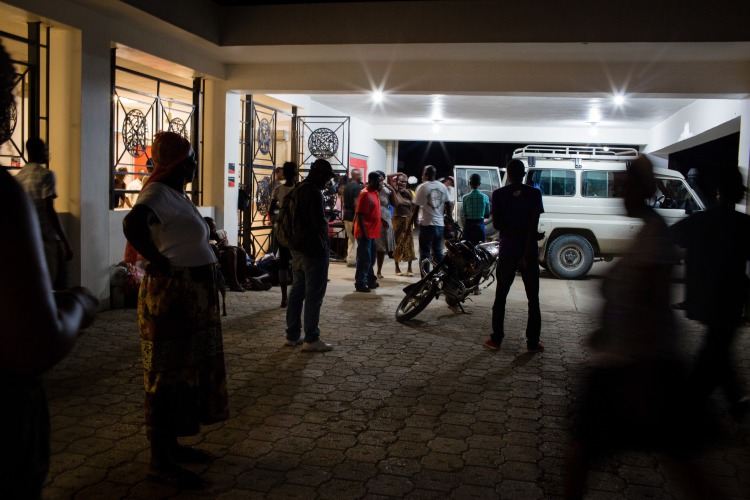
Photo: An ambulance arriving to the Hôpital Universitaire de Mirebalais emergency department with a transferred patient. In Haiti, social messaging facilitates interfacility transfers and patient care. Photo by Cecille Joan Avila/Partners In Health (used with permission)

## WHATSAPP TO IMPROVE HOSPITAL OPERATIONS AT HÔPITAL UNIVERSITAIRE DE MIREBALAIS

WhatsApp groups are essential to HUM’s hospital operations. HUM’s nursing leadership uses a WhatsApp group daily for hospital-wide bed management, adjusting staffing assignments, managing emergency situations, and disseminating general updates and communications. During mass casualty situations and when patient surges are anticipated – including the lead-up to Hurricane Matthew and during the fiercely-contested 2016 Haitian elections – WhatsApp groups with hospital leadership and departmental chairs facilitated communication and resource allocation through regular updates on patient numbers, available bed spaces, staffing and supply needs. Similarly, during a recent outbreak, a “diphtheria updates” WhatsApp group facilitated moving patients into isolation spaces and accelerated contact-tracing by simultaneously notifying community health and infection control teams of new cases.

Social messaging also improves operations for HUM’s emergency department (ED). Since Emergency Medicine (EM) is a new clinical specialty in Haiti, until recently the ED leadership paired senior Haitian staff and international emergency physicians (EPs) to build capacity. Since the international support was sometimes remote, constant and reliable communication was essential. An ED leadership WhatsApp group linked the four physician leaders and facilitated instantaneous communication, decision-making, and collaboration, while building local leadership capacity. The department has now successfully transitioned to all local leadership, who continue to use WhatsApp to facilitate ED operations. Additionally, an all-ED staff WhatsApp group transmits department news, policies, mass casualty events, security updates, and even birthday wishes, improving both operations and morale. Lastly, although e-consultation is not formalized, ED staff frequently send x-ray photos to orthopedic consultants, facilitating patient treatment and disposition.

## WHATSAPP TO IMPROVE EDUCATION DELIVERY AT HUM

For Haiti’s first EM residency, WhatsApp expands teaching opportunities. As a new specialty, few trained EPs are available to supervise EM residents. This initially posed a dilemma: should residents train only in the HUM ED, with EM attending supervision but without exposure to hospitals and pathology elsewhere in Haiti, or should residents rotate at affiliated hospitals without EM specialist supervision, putting trainees and patients at possible risk? Remote social messaging back-up offered a middle ground, allowing residents to gain experience at other sites while ensuring they could reach EP attendings with questions. We initially attempted video conferencing typical of telemedicine, but found Whatsapp more successful given its baseline popularity with residents and low band-width requirements.

Similarly, at night, WhatsApp telemedicine allows senior residents to gain experience working independently. Senior residents supervise juniors and run the department, but have an assigned attending remotely available. Via WhatsApp, residents send photos of EKGs and laboratory results, videos of exam findings and ultrasounds, and make free local and international phone calls over WiFi, allowing effective remote consultation. If needed, in-person back-up is available, but rarely required. Remote supervision allows limited faculty resources to stretch further and prepares residents to be independent clinicians and teachers after graduation.

To facilitate learning, all EM residents participate in a resident-led, faculty-guided WhatsApp group where interesting cases, teaching pearls, and questions are posted. Residents doing off-site rotations several hours away employ the feed for clinical questions and patient management advice (de-identified to protect privacy), and to stay in touch with their missed co-residents for support.

After a training program is completed, WhatsApp groups offer continuing education and support for participants. Graduates from HUM’s neonatal nurse intensive care training certificate work throughout Haiti, but can always turn back to a moderated WhatsApp group for advice. Similarly, international partners for HUM’s surgical team training use WhatsApp to provide ongoing distance coaching for participants after in-person trainings.

Finally, social messaging is not just beneficial for trainees. With few Haitian EPs, the EM residency at HUM relies on teaching and clinical supervision from visiting US and Canadian EPs. However, these attending physicians may be unfamiliar with the local context. Social messaging facilitates communication between these visiting physicians and our international leadership team, and strengthens the skills of junior US- and Canadian-trained faculty pursuing global health careers.

## WHATSAPP FOR INTER-HOSPITAL COMMUNICATION

At a national level, there is an ED communication group that includes prehospital groups and ED leadership at most major hospitals. This group helps with pre-hospital notification for large accidents and facilitates patient transfers between hospitals, allowing participants to reach multiple hospitals at once when searching for a hospital to accept a transfer, saving time and money.

## CONSIDERATIONS FOR SUCCESSFUL USE

As our experience in Haiti demonstrates, social messaging creates educational opportunities and facilitates clinical operations. As use expands into other clinical environments, best practices at each site will need to be developed. Any proposed role for messaging technology mandates caution and evaluation of risks and benefits to ensure responsible and appropriate use. We propose five considerations for deciding how and when to use social messaging in clinical care.

First, consider connectivity, cellular data costs, and smartphone availability in each setting. Most of our staff’s phones support WhatsApp, but this may not be true in all settings. Though social messaging requires WiFi or cellular phone internet access, we have found social messaging more available than email communications, particularly for staff without personal computers. Hospital WiFi connections, even when too slow for voice calls, typically allow social messaging. When WiFi is not available, using cellular data for WhatsApp text and calls is affordable due to small amounts of data required. Similarly, since clinical images can be gradually uploaded over slow connections, we find image resolution sufficient, unlike video chats which are frequently disrupted due to low-bandwidth.

Second, group social messaging feeds require active administration, including ‘rules of conduct’ and periodic membership review. Members must understand the group’s intent to facilitate clear communication. Groups for emergent or high level messages should be clear of casual conversation to avoid interrupting members unnecessarily. We find this requires periodic reminders by a designated senior administrator. Groups for non-emergent functions can be more loosely regulated, as members can mute the group and check it daily for updates. Further, someone should be assigned to manage group membership, ensuring its continued relevance as staff come and go. In larger groups, new members may require introduction beyond WhatsApp’s automatic notification that a member was added. Finally, when deciding group size, consider the group’s objective. We advise limiting membership to people who would be invited to an ‘in person’ meeting with the same purpose. Groups intended to broadly communicate information can be larger, whereas groups to facilitate decision-making should have the fewest possible members.

Third, assure patient privacy. Privacy is an essential ethical and legal consideration in all areas of health care, and social messaging should be no exception. For many services, information passes through and is stored unencrypted on a central server [13]. Though WhatsApp’s end-to-end encryption increases security by keeping messages on the central server private [14], messages are received on individual smart phones which may not be encrypted or password protected. Similarly, cloud backups of phone data are unencrypted. Given this, all patient information and photographs must be de-identified and photographs taken only with permission. This also protects privacy against lost or stolen devices.

Fourth, we recommend safeguards to prevent disseminating incorrect information. Social media can spread false information [15]; similar risks exist with social messaging. Though false information is more likely to come from simple error than deliberate deceit, educational feeds require a reliable faculty moderator. Since messages arrive at all hours, we have found having several faculty on a group ensures at least one person can correct information as needed.

Finally, we recommend backing cross-cultural remote consultation with in-person relationships. Our international faculty providing remote social messaging support also supervise residents in-person in the HUM ED. The pre-existing personal connections facilitate trust and communication necessary for effective teaching. Residents have a working relationship with each remote back-up attending, empowering them to text or call at all hours. Attendings know each resident’s abilities, and understand the system and context. These personal connections and contextual understanding are essential to our program’s success.

In addition to these five principles, we recommend users consider potential limitations to social messaging. First, consider patient perception of provider phone use and, if needed, develop usage guidelines. Second, the quality of remote supervision and consultation depends on what, how, and when information is provided; both sides may need training on this. Similarly, WhatsApp cannot replace in-person supervision for complex cases and procedures, but can create a false sense of security in these circumstances. Third, while we have found bandwidth adequate to gradually upload photos, we rarely require high resolution images; this may be different in some medical fields. Fourth, our experience has focused on nurses, physicians and hospital administrators; different strategies may be required for use with patients or other employees less likely to have smartphones. Finally, the evidence base for social messaging remains limited. Future studies should evaluate and quantify the impact of social messaging on hospital operations metrics, provider knowledge, clinical outcomes, and disaster response.

## CONCLUSION

Social messaging offers a powerful new medium for global medical education and health care operations, including daily hospital management, mass casualty response and disaster situations. For many programs in resource-limited settings, supervision, education, and support are constrained by gaps in human and monetary resources. Despite its limitations, social messaging offers a compelling opportunity to improve global health delivery and education. Our positive experiences in Haiti add to the growing evidence of the efficacy of this new modality, and we recommend its appropriate use to improve health equity.
